# Using community-owned resource persons to provide early diagnosis and treatment and estimate malaria burden at community level in north-eastern Tanzania

**DOI:** 10.1186/1475-2875-11-152

**Published:** 2012-05-03

**Authors:** Acleus S M Rutta, Filbert Francis, Bruno P Mmbando, Deus S Ishengoma, Samwel H Sembuche, Ezekiel K Malecela, Johari Y Sadi, Mathias L Kamugisha, Martha M Lemnge

**Affiliations:** 1 National Institute for Medical Research, Tanga Centre, P.O. Box 5004, Tanga, Tanzania

## Abstract

**Background:**

Although early diagnosis and prompt treatment is an important strategy for control of malaria, using fever to initiate presumptive treatment with expensive artemisinin combination therapy is a major challenge; particularly in areas with declining burden of malaria. This study was conducted using community-owned resource persons (CORPs) to provide early diagnosis and treatment of malaria, and collect data for estimation of malaria burden in four villages of Korogwe district, north-eastern Tanzania.

**Methods:**

In 2006, individuals with history of fever within 24 hours or fever (axillary temperature ≥37.5°C) at presentation were presumptively treated using sulphadoxine/pyrimethamine. Between 2007 and 2010, individuals aged five years and above, with positive rapid diagnostic tests (RDTs) were treated with artemether/lumefantrine (AL) while under-fives were treated irrespective of RDT results. Reduction in anti-malarial consumption was determined by comparing the number of cases that would have been presumptively treated and those that were actually treated based on RDTs results. Trends of malaria incidence and slide positivity rates were compared between lowlands and highlands.

**Results:**

Of 15,729 cases attended, slide positivity rate was 20.4% and declined by >72.0% from 2008, reaching <10.0% from 2009 onwards; and the slide positivity rates were similar in lowlands and highlands from 2009 onwards. Cases with fever at presentation declined slightly, but remained at >40.0% in under-fives and >20.0% among individuals aged five years and above. With use of RDTs, cases treated with AL decreased from <58.0% in 2007 to <11.0% in 2010 and the numbers of adult courses saved were 3,284 and 1,591 in lowlands and highlands respectively. Malaria incidence declined consistently from 2008 onwards; and the highest incidence of malaria shifted from children aged <10 years to individuals aged 10–19 years from 2009.

**Conclusions:**

With basic training, supervision and RDTs, CORPs successfully provided early diagnosis and treatment and reduced consumption of anti-malarials. Progressively declining malaria incidence and slide positivity rates suggest that all fever cases should be tested with RDTs before treatment. Data collected by CORPs was used to plan phase 1b MSP3 malaria vaccine trial and will be used for monitoring and evaluation of different health interventions. The current situation indicates that there is a remarkable changing pattern of malaria and these areas might be moving from control to pre-elimination levels.

## Background

Malaria remains a public health problem despite the current reports which indicate that there has been a decline of malaria burden in various parts of sub-Saharan Africa [1,2]. The changing pattern of malaria has been attributed to the wide use of insecticide-treated nets (ITNs), indoor residual spraying (IRS), availability of prompt diagnosis and treatment with effective anti-malarial drugs, the artemisinin combination therapy (ACT). However, misdiagnosis and delays to seek treatment have been identified to be among the major causes of death due to malaria. To address these shortfalls, the Roll Back Malaria Initiative recommends early case management based on early diagnosis and treatment within 24 hours of onset of initial symptoms of malaria, as the best strategy for malaria control by halting the progression of malaria infection to severe form of the disease and deaths [3].

In sub-Saharan Africa, where health resources are limited, fever has been used as a cornerstone for instituting presumptive treatment of malaria [4-6] and it is an important component of the guidelines for integrated management of childhood illnesses (IMCI) [7] and home management of malaria [8]. However, with a declining malaria burden in some areas in Africa, dependency on fever as a marker of malaria infection may be compromised by altered sensitivity and specificity due to low levels of malaria prevalence in the community [4,9]. Furthermore, with most sub-Saharan countries changing to ACT as first-line anti-malarials, it is imperative that confirmation of malaria parasites is done before prescribing these expensive drugs, as recommended by the World Health Organization (WHO) [10]. Case treatment based on laboratory tests will potentially reduce over-use of ACT, costs of case management, drug pressure and thus delay the emergence of drug resistance. However, in low-resourced countries, case management based on laboratory diagnosis is difficult to realize due to unavailability of laboratory facilities, including skilled microscopists [11]. Therefore, simple but effective innovative options for malaria management should be devised, especially in rural settings with limited access to laboratory services.

In a recent study on malaria epidemiology, Mmbando *et al.*[12] observed a lower prevalence of malaria and anaemia in the villages where community-owned resource persons (CORPs) were offering early diagnosis and treatment of malaria. In CORPs strategy, minimally trained community health workers have been used for the management of uncomplicated malaria using first-line anti-malarial drugs based on fever as a guide to start treatment [13]. Furthermore, Hopkins *et al.*[8] reported that when health workers with minimal training are used at community level, they can be useful for the provision of early diagnosis and treatment of malaria and collection of epidemiological data. Thus, a system for passive case detection of fever was initiated in 2006 in four villages (two in the lowlands and two in the highlands) in Korogwe district, north-eastern Tanzania, using CORPs to provide early diagnosis and treatment of malaria, and collect data for estimation of malaria burden. The information obtained was used in planning phase 1b MSP3 malaria vaccine trial and will be used for monitoring other health interventions.

## Methods

### Study area and population

The study was conducted in Korogwe district, north-eastern Tanzania. The district is located between latitudes 4 15’ and 5 15’S, and longitudes 38 0 and 38 45’E. The district profile and details of the study area have been described elsewhere [12,14]. The study involved four villages (Mng’aza and Kwashemshi in the lowland stratum, and Kwamhanya and Magundi in the highland stratum) which are part of 14 villages under Korogwe Demographic Surveillance System (DSS) [14]. In this part of Tanzania, lowlands are characterized by high malaria compared to highlands which have low malaria transmission [12,15-18].

### Selection of study villages, recruitment, training and supervision of community-owned resource persons (CORPs)

The study villages were selected in October 2005 as described elsewhere [12,14]. Thereafter, meetings were held with village members to seek community ascent and engagement as previously described [19]. Suitable candidates for the posts of community-owned resource persons (CORPs) that met specified criteria, including at least primary/secondary education and good relationship with community members, were recruited with the support of village leaders of the study villages. Individuals recruited received monthly payments based on the National Institute for Medical Research (NIMR) staff regulations to compensate for their time.

The selected candidates were trained in November 2005 in basic skills for the provision of early diagnosis and treatment of fever cases with first-line anti-malarials. After the training, they were deployed to their respective posts and provided with standard operating procedures (SOPs) and guidelines developed in the national language of Kiswahili. The SOPs and guidelines provided simple and clear instructions on how to complete a morbidity questionnaire: by taking records of body weight, patients’ history, body temperature (using a digital thermometer) and collecting finger prick blood for preparation of blood smears. In addition, CORPs were given instructions on how to perform a rapid diagnostic test (RDT) for detection of malaria parasites, provide treatment for malaria, and referrals to the next level of health care for cases they could not handle. Further retraining of CORPs was done in December 2006 just before the introduction of RDTs and artemether-lumefantrine (*AL*).

Weekly supervision of COPRs' activities was done by a clinician and a laboratory technician from Tanga Centre. During supervision, the team discussed with CORPs their performance and assessed the progress of their activities in order to rectify any anomaly. Actions taken and on-job training support provided to CORPs were recorded on the supervision checklist. All essential supplies and medications were delivered to CORPs during supervision. A checklist was used to assess the performance of CORPs on three main criteria, including filling of morbidity questionnaire, organization of working areas (including arrangement of working tools, handling of SOPs and waste disposal) and preparation of blood smears for detection of malaria parasites. Scores ranging from 1 (poor) to 4 (excellent) were used to rank the performance of CORPs on different items under the above broad categories and the maximum score that could be attained was 44.

### Malaria case management

All cases attended by CORPs had morbidity questionnaires completed and blood smears taken for parasite detection by microscopy, which was done later in the laboratory at NIMR - Tanga Centre. For treatment of febrile cases, kits with first-line anti-malarials, paracetamol, and other basic items were supplied to CORPs. Treatment was done with first-line anti-malarials based on the national guidelines for treatment of uncomplicated malaria [20,21]. Sulphadoxine-pyrimethamine (SP) was used until January 2007 while *AL* was introduced in February 2007.

From January 2006 to January 2007, all individuals reporting to CORPs were presumptively treated with SP. Following introduction of *AL*, together with RDTs, all under-fives were treated with *AL* irrespective of RDT results, while those aged five years and above received treatment only if they had positive RDTs. RDTs employed were Paracheck® or Parahit®, as described previously [22]*.* Blood smears were collected during weekly supervision and processed later in the laboratory as described elsewhere [22]. Referrals were made to health facilities, including Korogwe district hospital, for further investigations and management of cases that could not be handled by CORPs.

### Anti-malarial consumption pattern

In the absence of RDTs, all cases with a history of fever in the past 24 hrs or fever (axillary temperature of ≥37.5°C) at presentation attended by CORPs were given medication based on clinical diagnosis. To determine the anti-malarial dispensing pattern before and after introduction of RDTs, the number of cases that would have been treated in the absence of RDTs and the actual number treated based on RDT results, were compared. The number of anti-malarial tablets saved was calculated by subtracting the actual number of tablets dispensed from those that would have been dispensed in the absence of RDTs. Adult courses of anti-malarials saved were obtained by dividing the number of tablets saved by 24, which is a full dose of *AL* for individuals weighing ≥35 kg. The number of cases treated in 2006 (before introduction of RDTs and *AL*) was used as a reference.

### Data management and analysis

Data were managed using Microsoft Access software and were double entered for the purpose of validation and the analysis was carried out using STATA version 11 (STATA Corp Inc., TX, USA). Malaria incidence rate was computed by dividing the number of positive cases by person years of follow-up. The person years of follow-up time was calculated using data collected by the Korogwe DSS. Proportions were compared by Chi-square test and Poisson regression was used to compare the incidence of malaria across age groups and strata. *P*-value < 0.05 was considered significant.

### Ethical consideration

This study was conducted as part of a larger project, which received ethical clearance from the Medical Research Coordination Committee (MRCC) of NIMR. The study protocol was presented and discussed with the district authorities, and community engagement and participation were conducted as described elsewhere [19]. Individual cases and parents/guardians of children attended by CORPs were asked for oral and written informed consent which was signed and documented on a small section located on the top of the morbidity questionnaire.

## Results

### Malaria case management

During the five years of follow-up, a total of 15,729 cases were attended by CORPs in both lowland (10,585) and highland (5,144) villages. Of these, 4,766 (30.3%) had fever (axillary temperature ≥37.5°C) at presentation. Children aged less than five years accounted for 23.1% of all cases (25.4% and 18.2% from the lowlands and highlands, respectively). The proportion of under-fives increased significantly over the study period in both lowlands and highlands (*p* < 0.001, Table 1). Under-fives with fever at presentation were 1,286 (47.8%) from lowland and 417 (44.7%) from highland villages.

**Table 1:** Characteristics of cases attended by CORPs from 2006 to 2010

**Year**						
Variable	2006	2007	2008	2009	2010	Test statistics
**Lowland**						
Number of cases	1701	2986	2795	1803	1300	
Children <5 years (n, %)	376(22.1)	680(22.8)	712(25.5)	566(31.4)	357(27.5)	χ² = 39.5, *p* <0.001
Age in years (median, IQR)	20.7	15.2	12.8	11	11.3	
	(6.1-43.9)	(5.6-38.4)	(4.9-33.1)	(4.0-30.7)	(4.4-33.0)	
Malaria parasite positive (n, %)	406(23.9)	1052(35.2)	667(23.9)	112(6.2)	86(6.6)	χ² = 465.5, p <0.001
Fever (≥37.5°C, n, %)	476(28.0)	1014(34.0)	858(30.7)	516(28.6)	421(32.4)	χ² = 0.26, *p* = 0.613
GM Pf density (rings/μl, 95%CI)	1980	2367	2518	667	3714	F = 6.1, *p* < 0.001
	(1481–2680)	(1973–2853)	(2020–3159)	(406–1139)	(2047–7060)	
**Highland**						
Number of cases	1406	1432	991	755	560	
Children <5 years (n, %)	213(15.1)	249(17.4)	165(16.6)	165(21.9)	142(25.4)	χ² = 30.7, *p* <0.001
Age in years (median, IQR)	21.2	16.6	14.6	15	13.6	
	(8.3-40.8)	(7.4-39.1)	(7.4-32.3)	(6.1-31.0)	(5.0-31.1)	
Malaria parasite positive (n, %)	137(9.7)	499(34.9)	166(16.8)	44(5.8)	36(6.4)	χ² = 49.3, *p* <0.001
Fever [≥37.5°C, (n, %)]	366(26.0)	456(31.8)	312(31.5)	208(27.6)	139(24.8)	χ² = 0.02, *p* = 0.892
GM Pf density (rings/μl, 95%CI)	957	2721	3740	5583	11629	
	(607–1558)	(2071–3611)	(2391–6000)	(2437–13965)	(6550–21438)	

**Figure 1:**
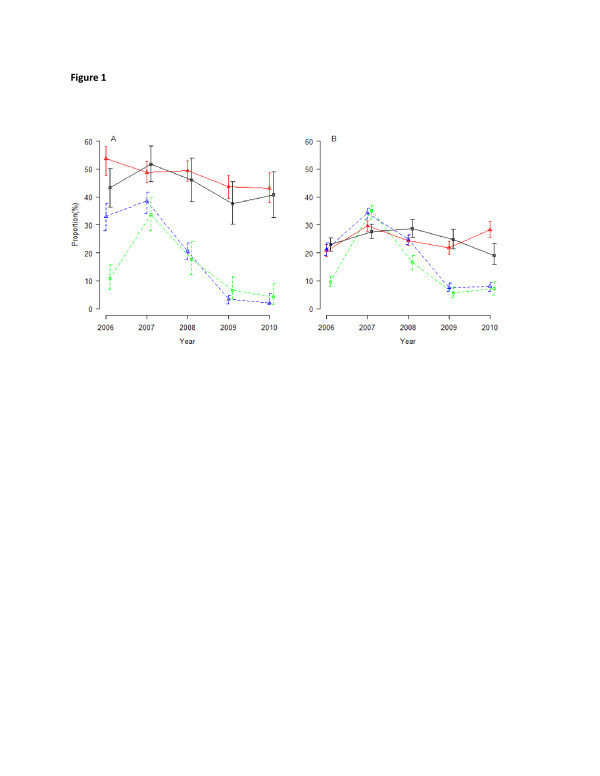
**Proportions of cases with fever and malaria parasites among under-fives (a), and individuals aged five years and above (b) in both lowland and highland villages between 2006 and 2010.** Solid lines represent fever cases (red, with triangles in lowlands and grey, with squares in highlands) and dashed lines for malaria parasite positive (blue, with triangles in lowlands and green, with squares in highlands).

Based on microscopy, 20.4% of the cases attended were positive for malaria parasites; and this was significantly higher in the lowlands (22.0%) compared to the highlands (17.2%, *p* < 0.001). Both the total number of cases attended and the proportion with malaria parasites declined over the study period (Table 1). A remarkable decline of slide positivity rate (more than 72.0%) was observed from 2008 and remained at <10%. The slide positivity rates were similar in the lowlands and highlands from 2009 onwards (Table 1 and Figure 1). However, the proportion of cases with fever at presentation declined slightly, but remained at >40% in under-fives and >20% among individuals aged five years and above throughout the study period (Figure 1a and b). Among cases with fever at presentation, only 33.4% had malaria parasites. The risk of developing fever was 3.3 times higher for individuals with malaria parasites compared to those without parasites after adjusting for the effect of strata, age and year of follow up (*p* < 0.001).

### Anti-malarial drug consumption, referrals and performance of CORPs

In 2006, over 97.0% of the cases aged five years and above, attended by CORPs were presumptively treated with SP (Table 2). After introduction of RDTs, the proportion of cases treated with *AL* decreased from <58.0% to <11% between 2007 and 2010 in both lowlands and highlands. The drop in the proportion of cases treated with *AL* increased consistently across the years (*p* < 0.001). The highest drop was seen in 2009 in the lowlands and 2010 in the highlands. Between 2007 and 2010, the total number of *AL* tablets saved in the lowland villages was 78,804 and in the highlands were 38,190 (Table 2), which translates to 3,284 and 1,591 adult courses respectively.

**Table 2:** Number of cases aged five years and above treated with anti-malarials before and after introduction of RDTs

**Year and Number attended (n)**	**Number would have been treated (%)**	**Actual number treated (%)**	**Percentage drop in dispensing (95% CI)**	**Number of tablets saved**
**Lowlands**				
2006(n = 1325)	1,285(97)	1,287(97.1)	−0.2	
		(−1.4 - 1.1), *p* = 0.818	(−1.4 - 1.1), *p* = 0.818	
2007(n = 2306)	2,087(90.5)	1,186(51.4)	39.1	23,940
		(36.7 - 41.4), *p* < 0.001	(36.7 - 41.4), *p* < 0.001	
2008(n = 2083)	1,819(87.6)	640(30.8)	56.8	23,742
-	-	(54.3 - 59.2), *p* < 0.001	(54.3 - 59.2), *p* < 0.001	
2009(n = 1237)	1,010(81.7)	100(8.1)	73.6	18,300
		(71.0 - 76.3), *p* < 0.001	(71.0 - 76.3), *p* < 0.001	
2010(n = 944)	713(75.5)	67(7.1)	68.4	12,822
		(65.2 - 71.6), *p* < 0.001	(65.2 - 71.6), *p* < 0.001	
**Highlands**				
2006(n = 1193)	1,187(99.5)	1,188(99.6)	−0.08	
		(−0.6 - 0.4), *p* = 0.762	(−0.6 - 0.4), *p* = 0.762	
2007(n = 1183)	1,095 (92.6)	680(57.5)	35.1	13,050
		(31.9 - 38.3), *p* < 0.001	(31.9 - 38.3), *p* < 0.001	
2008(n = 826)	697(84.4)	229(27.7)	56.7	9,846
		(52.7 - 60.6), *p* < 0.001	(52.7 - 60.6), *p* < 0.001	
2009 (n = 590)	528(89.6)	107(18.2)	71.5	8,754
		(67.5 - 75.4), *p* < 0.001	(67.5 - 75.4), *p* < 0.001	
2010(n = 418)	355(84.9)	45(10.8)	74.2	6,540
			(69.6 - 78.7), *p* < 0.001	

% = percent and 95% CI = 95% confidence intervals.

During the five years of follow-up, a total of 1,486 cases (including 168 under-fives, 11.3%) were referred to heath facilities, including Korogwe district hospital. Most referrals (75% of all cases and 88% of the under-fives) were from lowlands. The number of referrals increased from 2.4% in 2006 to 16.2% in 2010.

The performance of CORPs was assessed once weekly from the second week of March 2006 and covered a total of 250 weeks up to December 2010. Of the four CORPs assessed (one from each of the study villages), the mean number of assessments was 244 weeks (ranged from 241 to 247 weeks). The mean overall score of CORPs was 94.3% (range, 75.0% to 100%). The highest mean score was 95.2% in 2007 while the lowest was 93.6% in 2010. For each of the CORPs, the highest overall score was 96.1% while the lowest was 93.3%.

### Malaria incidence

Overall, a total of 2,858 episodes of malaria were detected in an at-risk population of 29,301 person years (PY) from both strata, between 2006 and 2010, giving an incidence rate of 98 per 1,000 PY. The overall incidence rate was 113 and 71 per 1,000 PY in the lowland and highland villages respectively. After adjusting for the effect of year, the incidence rates were significantly higher in lowlands by 32% (95% CI: 27–37, *p* < 0.001) compared to highlands. Generally, malaria incidence increased between 2006 and 2007, thereafter decreased consistently across the years in all age groups and in both strata (Table 3). A remarkable decline in malaria incidence was observed between 2008 and 2009. Between 2006 and 2008, the highest incidence of malaria was observed in children aged nought to nine years in both lowland and highland villages, but progressively shifted to individuals aged 10–19 years from 2009 onwards (Table 3).

**Table 3:** Age-specific malaria incidence rate per 1,000 person years

Age group/year	2006	2007	2008	2009	2010
	Cases	Cases	Cases	Cases	Cases
	[Incidence (95% CI)]	[Incidence (95% CI)]	[Incidence (95% CI)]	[Incidence (95% CI)]	[Incidence (95% CI)]
**Lowlands**					
0-4 yrs	121	272	152	20	7
	[206(173–245)]	[358(318–403)]	[185(158–217)]	[37(24–58)]	[11(5–24)]
5-9 yrs	101	252	170	30	17
	[192(158–234)]	[428(378–484)]	[267(230–311)]	[56(39–80)]	[31(19–50)]
10-19 yrs	79	205	148	49	27
	[98(79–125)]	[244(213–280)]	[169(144–199)]	[67(50–88)]	[35(24–51)]
20 + yrs	74	216	138	18	6
	[46(37–58)]	[124(108–141)]	[71(61–85)]	[12(7–18)]	[4(2–8)]
**Highlands**					
0-4 yrs	27	79	34	4	2
	[77(53–113)]	[189(151–236)]	[77(55–108)]	[12(11–24)]	[5(1–20)]
5-9 yrs	19	123	39	6	5
	[67(43–106)]	[390(326–464)]	[120(87–163)]	[22(17–27)]	[18(7–42)]
10-19 yrs	32	102	44	15	9
	[65(46–93)]	[199(164–241)]	[84(62–112)]	[33(20–55)]	[20(10–38)]
20 + yrs	33	134	31	14	10
	[35(25–49)]	[130(109–154)]	[29(21–42)]	[16(9–26)]	[10(6–19)]

Yrs = Years and 95% CI = 95% confidence intervals.

## Discussion

In most malaria endemic areas, diagnostic facilities are commonly unavailable and fever has, for a long time, been used to initiate anti-malarial treatment [23,24]. However, the applicability of fever as a point to start treatment for malaria might be problematic [23], mainly due to the recent introduction of artemisinin combination therapy (ACT), which is expensive compared to drugs which were commonly used in the past [10,25]. Furthermore, reports of changing patterns of malaria epidemiology with low burden of the disease in areas which were holo/hyperendemic to malaria, imply that correct diagnosis of malaria is critical to prevent over-diagnosis and over-treatment with ACT [8]. Misdiagnosis and delays to seek treatment have also been identified among the main causes of deaths due to malaria [13]. The Roll Back Malaria Initiative recommends early case management, based on early diagnosis and treatment within 24 hours of onset of initial symptoms of malaria, as the best strategy to halt the progression of malaria infection to severe form of the disease [3].

This study was initiated in 2006 in four villages with varying malaria transmission in Korogwe district, north-eastern Tanzania, using community-owned resource persons (CORPs) to provide early diagnosis and treatment of malaria, and collect data for estimation of malaria burden. The findings show that about 80% of the cases attended by CORPs over a period of five years had no malaria parasites. However, cases with fever among under-fives and those aged five years and above remained high throughout the study period despite decreasing slide positivity rates. The low slide positivity rates could be due to the declining malaria burden in the study areas [26]. Furthermore, cases attended by CORPs did not decline as for the slide positivity rates, thus most of the fevers could possibly be due to other infections [27-30]. Studies conducted in this and other areas of north-eastern Tanzania have shown low slide positivity rates for malaria and bacterial infections among children with severe febrile illnesses, suggesting that the causes of most fevers are not clearly known [28-32] (and C Mahende, pers. comm.).

In order to minimize over-treatment with expensive ACT, WHO recommends that the drugs should be prescribed to laboratory-confirmed cases only, using microscopy or RDTs [10]. In areas without laboratory capacity and with limited access to health facilities, RDTs remain the only option for malaria diagnosis. Introduction of RDTs in the study villages resulted in a significant reduction in consumption of *AL* among cases aged five years and above, both in the lowlands and highlands. Since the number of cases with fever among under-fives remained high, these were treated with *AL* based on IMCI guidelines irrespective of RDT results, leading to unnecessary use of the drugs by treating majority of cases with slide negative results. Despite low accuracy of RDTs used in this study [22], their use by CORPs reduced over-treatment and thus consumption of *AL*.

The increase in malaria incidence reported between 2006 and 2007, which was also associated with high slide positivity rates, was possibly due to abnormal and extended short rains towards the end of 2006. The consistent decrease of malaria incidence in both lowlands and highlands from 2008 onwards, indicates that the burden of malaria has indeed declined as reported elsewhere [26]. Furthermore, the results revealed that the slide positivity rates were similar (≈6%) in both lowland and highland villages from 2009 onwards. Ongoing studies in these villages have also shown that the slide positivity rates further declined, reaching 5.9% in the lowland and 1.2% in highland villages by December 2011 (F Francis, pers. comm.). However, previous studies conducted in the same and nearby areas had shown that highlands had low malaria transmission compared to lowlands [12,33,34]. The current situation indicates that there is a remarkable changing pattern of malaria and these areas might be moving from control to pre-elimination levels [35].

A progressive shift of malaria incidence from children under 10 years of age to those aged 10–19 years observed from 2009 onwards could possibly be due to low exposure to infections and delayed development of anti-malarial immunity [36,37]. Previous studies conducted in lowland and highland areas of north-eastern Tanzania, reported that the highest burden of malaria involved under-fives while the peak parasite prevalence in areas of high malaria transmission occurred in children aged five to nine years [38]. Similar findings have been reported in other malaria endemic areas [39], and it is attributed to early development of acquired immunity resulting from early exposure to malaria infections. The current shift of malaria burden to older individuals further indicates that the burden of malaria has significantly declined leading to low and delayed development of acquired immunity in the local population. This suggests that individuals from these areas might be highly susceptible to malaria outbreaks. Thus, more efforts are needed to sustain these achievements and reduce the impact of malaria outbreak, which could potentially occur.

The overall performance was good and did not differ among the CORPs, during the five years of follow-up. This could be attributed to the support that was consistently given to CORPs during supervision, whereby individual performance was assessed and problems were discussed and resolved. As shown elsewhere, CORPs in this study had high performance on malaria diagnosis using RDTs and adherence to test results [22]. Studies conducted elsewhere have shown that proper training and adequate supervision of community health workers and other health staff are critical and can potentially lead to high level of performance in different programmes, including home management of febrile cases [40]. Increased number of cases referred to health facilities after introduction of RDTs and *AL* might be due to adherence of CORPs to RDT results whereby individuals aged five years and above with negative tests were not given anti-malarials. Although CORPs were able to refer patients to health facilities as directed by SOPs, follow-up of referred cases was not done due to the limitations associated with the design of this study.

## Conclusions

This study showed that when provided with basic training, supervision and simple diagnostic tools, CORPs can provide early diagnosis and treatment at community level, and therefore reduce consumption of anti-malarials. The progressive decline of malaria incidence and slide positivity rates among cases of all age groups in both lowland and highland villages, suggests that treatment guidelines need to be urgently revised so that all cases can be tested with RDTs before treatment is given. Given the number of cases with fever reporting to CORPs has not declined despite low burden of malaria, further surveillance by passive case detection (PCD) of febrile illnesses is still needed in order to provide an opportunity to identify other sources of fever in the study communities. Furthermore, data collected by CORPs can be used for monitoring and evaluation of different interventions and millennium development goals (MDGs) related to malaria.

## Competing interests

The authors declare that they have no competing interests.

## Authors’ contributions

ASMR, BPM, DSI, MLK and MML participated in the design and supervised the overall implementation of the study. BPM and FF were responsible for management and analysis of the data. SS, EKM, JYS were involved in training and supervision of CORPs and EKM and JYS supervised reading of blood smears by microscopy. ASMR, BPM, FF, DSI, MLK and MML wrote the manuscript. All authors read and approved the final version of the manuscript.
